# Integrated Maternal Care Strategies in Low- and Middle-Income Countries: A Systematic Review

**DOI:** 10.5334/ijic.6254

**Published:** 2022-06-22

**Authors:** Laura van der Werf, Silvia Evers, Laura Prieto-Pinto, Daniel Samacá-Samacá, Aggie Paulus

**Affiliations:** 1 Department of Health Services Research, Care and Public Health Research Institute (CAPHRI), Faculty of Health Medicine and Life Sciences, Maastricht University, P.O. Box 616, 6200 MD Maastricht, NL; 2 Centre for Economic Evaluation and Machine Learning, Netherlands Institute of Mental Health and Addiction, Trimbos Institute, Utrecht, NL; 3 MSc Clinical Epidemiology, Universidad Nacional de Colombia, CO; 4 MSc Clinical Epidemolgy student, Universidad Nacional de Colombia, CO

**Keywords:** integrated care, maternal health services, integrated delivery of health care, continuity of patient care, maternal-child health services, care coordination

## Abstract

**Background and aim:**

Ineffective organisation of care leads to increased morbidity and mortality in neonates and their mothers. We aimed to identify and describe strategies used in low- and middle-income countries that attempt to deliver coherent, coordinated, and continuous services (i.e., integrated care) and how the various strategies affect the organisation of care.

**Methods:**

We conducted a systematic literature review to identify, appraise, and synthesise relevant evidence about strategies for integrating maternal care in low- and middle-income countries, searching multiple electronic databases.

**Results:**

Fourteen studies met our inclusion criteria. We identified five types of integration strategies: 1) organisational, 2) service/professional, 3) functional, 4) organisational combined with normative strategies, and 5) clinical combined with functional integration strategies. The most frequent types of strategies were organisational, and service/professional integration strategies. We did not identify any publications describing systemic integration strategies implemented in low- and middle-income countries.

**Conclusions:**

Most types of strategies described in theory have been implemented and studied in low- and middle-income countries. Our findings suggest that different types of strategies may lead to comparable organisational outcomes. For example, organisational integration strategies and professional or service integration strategies may similarly influence inter-organisational collaboration. Inter-organisational collaboration may play a particularly important role in the context of maternal care integration.

## Background and introduction

According to the World Health Organisation, all women should have access to maternal health services. These include care during pregnancy (prenatal care), skilled care during childbirth (perinatal care), and care and support during the weeks after childbirth (postnatal care) [1,2]. For these maternal services to successfully reduce maternal morbidity and mortality as well as for patients to have an adequate experience of care, the process of care must be adequately organised. This means that the tasks that are part of the process of care must be departmentalised and assigned so that they can be performed in a way that successfully produces the desired result [3]. However, often women perceive maternal care to be disjointed, uncoordinated, and incoherent, particularly in low- and middle-income countries [4,5,6,7,8].

The experience of disjointed care stems from an inadequately organised care process (i.e., how the process of care is divided into distinct tasks and the way coordination among these is achieved [9,10]). A care process in which there is a systemic misalignment of incentives and a lack of coordination that generates an inefficient allocation of resources and may harm patients is known as fragmented care [11]. Previous research has established that fragmentation of maternal care decreases its quality, increasing neonatal and maternal morbidity and mortality and generating unsatisfying work conditions for healthcare professionals [12,13,14,15,16].

As a response to fragmentation in maternal care, integrated care, a broad concept that encompasses the delivery of coherent, coordinated, and continuous services by cooperating actors across the care continuum [17,18], has been proposed as a possible solution for improving the organisation of this type of care and consequently for improving its quality [19].

Integration of care can be achieved through various strategies (i.e., plans of action designed to achieve an overall long-term aim, in this case, providing integrated care). *Organisational integration strategies* are those in which coordination of care is achieved through inter-organisational relationships (e.g., contracting, strategic alliances, knowledge networks, mergers) [17,20,21]. *Functional integration strategies* are those in which non-clinical support and back-office functions are integrated, for example, through the use of shared electronic medical records and shared financing and management [17,20,21]. *Service/professional integration strategies* create new inter-professional partnerships and ways of interaction based on shared competencies, roles, responsibilities, and accountability (e.g., multidisciplinary teams, periodic meetings between interrelated professional groups or departments)[20]. *Clinical integration strategies* aim to coordinate care through understanding it as a single process that crosses intra- and inter-professional boundaries and organisational boundaries [20,21,22] (e.g., shared clinical practice guidelines and pathways). *Normative integration strategies* coordinate organisations, professional groups, and individuals through a common frame of reference (i.e., shared mission, vision, values, and culture) [20,21,22]. Finally, *systemic integration strategies* integrate care through coherent rules and policies at the various levels of organisations [17,21]. A summary of the definitions of integration strategies is provided in Table 1.

**Table 1:** Types of care integration strategies and definitions.


TYPE OF STRATEGY	DEFINITION

*Organisational integration strategies*	Strategies aimed at achieving coordination of care through inter-organisational relationships (e.g., contracting, strategic alliances, knowledge networks, mergers) [17,20,21].

*Functional integration strategies*	Strategies aimed at integrating non-clinical support and back-office functions, for example, through the use of shared electronic medical records and shared financing and management [17,20,21].

*Service/professional integration strategies*	Strategies aimed at creating new inter-professional partnerships and ways of interaction based on shared competencies, roles, responsibilities, and accountability (e.g., multidisciplinary teams, periodic meetings between interrelated professional groups or departments) [20].

*Clinical integration strategies*	Strategies aimed at achieving coordination of care through understanding it as a single process that crosses intra- and inter-professional boundaries and organisational boundaries (e.g., shared clinical practice guidelines and pathways) [20,21,22].

*Normative integration strategies*	Strategies aimed at achieving coordination of care between organisations, professional groups, and individuals through a common frame of reference (i.e., shared mission, vision, values, and culture)[20,21,22].

*Systemic integration strategies*	Strategies aimed at Integrating care through coherent rules and policies at various levels of organisations [17,21].


Previous research has identified five care features that are critical in assessing whether care is adequately organised to provide integrated care [23,24]. The critical features identified are the coordination of the care process, patient-focused organisation of care, communication with patients and family, collaboration with primary care, and follow-up of the care process [23,24].

*Coordination of the care process* refers to a process in which team members make concrete agreements; team members are familiar with the various steps in the care process, and there is an optimum timing of activities within the care process. A *patient-focused organisation of care* refers to having the patient’s needs and preferences at the centre of the process of organising care. *Follow-up of the care* process refers to monitoring variances, risks of complications, and outcomes - including patient satisfaction - throughout the complete care process. *Communication with patients and family* refers to a process in which time is provided explicitly to listen and provide information to patients and family members. Furthermore, patients are asked explicitly for their consent regarding the proposed care [24].

*Inter-organisational collaboration* is the successful cooperation among different organisations that makes task achievement possible through united effort [25]. A critical subtype of inter-organisational collaboration is *collaboration with primary care*. This type of inter-organisational collaboration refers to care processes in which centres which provide highly complex health care consider primary care to be an equal partner, and good cooperation exists between different levels of care [24].

An additional important concept linked to the assessment of care organisation is interprofessional collaboration. *Interprofessional collaboration* is “the process by which different health and social care professional groups work together to positively impact care. Interprofessional collaboration involves regular negotiation and interaction between professionals, which values the expertise and contributions that various healthcare professionals bring to patient care” [26]. Likewise, effective *inter-organisational collaboration* is the successful cooperation among different organisations that makes task achievement possible through united effort [25]. A summary of organisational outcomes can be found in Table 2.

**Table 2:** Organisational outcomes and definitions.


ORGANISATIONAL OUTCOME	DEFINITION

*Coordination of the care process*	Refers to a process in which team members make concrete agreements, team members are familiar with the various steps in the care process, and there is an optimum timing of activities within the care process [24].

*Patient-focused organisation of care*	Refers to having the patient’s needs and preferences at the centre of the process of organising care [24].

*Follow-up of the care* process	Refers to monitoring variances, risks of complications, and outcomes – including patient satisfaction - throughout the complete care process [24].

*Communication with patients and family*	Refers to a process in which time is provided explicitly to listen and provide information to patients and family members. Furthermore, patients are asked explicitly for their consent regarding the proposed care [24].

*Inter-organisational collaboration*	The successful cooperation among different organisations that makes task achievement possible through united effort. A critical subtype of inter-organisational collaboration is *collaboration with primary care*. This type of inter-organisational collaboration refers to care processes in which centres which provide highly complex health care provision consider primary care to be an equal partner, and good cooperation exists between different levels of care [25].

*Interprofessional collaboration*	Defined as “the process by which different health and social care professional groups work together to positively impact care. Interprofessional collaboration involves regular negotiation and interaction between professionals, which values the expertise and contributions that various healthcare professionals bring to patient care” [26].


Although a systematic literature review recently studied strategies for coordinating maternal health services implemented in the United States [27], and in 2007 a literature review that included studies from the United States, Canada and Europe regarding integrated perinatal care was conducted [28], little is known about the integration strategies of maternal health services implemented in low- and middle-income countries. Furthermore, looking at the current literature, the effect these strategies ultimately have on the organisation of care, as perceived by women, physicians, nurses, allied health professionals, and managers, remains unclear [29]. Integration strategies for maternal care in low and middle-income countries are especially relevant because these countries have a more significant burden of maternal and neonatal morbimortality [29], both related to the fragmentation of maternal care [12,13,14,15,16]. Moreover, the health systems of low- and middle-income countries have particularities that relate to the more pronounced scarcity of resources and infrastructure deficiencies [30]. It is important to know the types of strategies used for integrating maternal care in these countries, as well as the effect they have had on integration of care. This knowledge can guide the development and implementation of strategies for successfully reaching the goal of changing the way maternal care is organised to provide integrated care (i.e., which strategies are effective for integrating care). Hence, this study aimed to identify and describe the strategies that have been used in low- and middle-income countries to integrate maternal care (i.e., deliver coherent, coordinated, and continuous services by cooperating actors across the care continuum) and their effect on the organisation of care.

## Methods

We conducted a systematic literature review to identify, appraise, and synthesise all the relevant evidence about strategies for integrating maternal care in low- and middle-income countries. It was performed following the recommendations of the Cochrane Handbook for Systematic Reviews and reported according to Preferred Reporting Items for Systematic Reviews and Meta-Analyses [31,32]. We followed a previously developed protocol registered in PROSPERO (CRD42020160718). We searched the following databases and search engines for relevant publications: MEDLINE, Embase, JSTOR, Healthcare Administration Database (ProQuest), Web of Science, Google Scholar, and Scopus. MEDLINE and EMBASE were selected because they are the most comprehensive databases indexing research related to health care. JSTOR, Scopus, and Web of science were selected because healthcare service research is a multidisciplinary field, and these databases and search engines allow access to information from multiple disciplines [33,34,35]. We included the Healthcare Administration Database because of its emphasis on relevant fields for care integration strategies such as business management, personnel management, health economics, and public health administration [36]. Finally, we used Google Scholar for increasing the search’s comprehensiveness, as it includes between 2 and 100 million records of academic and grey literature [37]. The search strategies were structured using Boolean, truncation, and proximity operators adapted for each search engine. We used both free text and controlled vocabulary. Such terms as the following were included in the search strategies: ‘mothers’, ‘maternal health’, ‘maternity’, ‘motherhood’, ‘maternal-child health centres’, ‘maternal health services’, ‘prenatal care’, ‘perinatal care’, ‘postnatal care’, ‘prenatal care’, ‘antenatal’, postpartum ‘, ‘obstetric care’, ‘integrated care’, ‘coordinated care’, ‘disease management’, ‘case management’, ‘low income countries’, ‘middle income countries’, ‘middle income economies’, ‘lower-middle-income economies’, ‘less developed countries’, ‘developing countries ‘under-developed countries’, and ‘less developed economies’. Details of the search strategies are presented in Appendix 1.

We screened the references of selected publications to identify other relevant publications (snowballing). We identified other publications citing the selected papers (reverse snowballing) through the citation tracking facility found in Google Scholar, WEB of knowledge, and PubMed (MEDLINE). Furthermore, we performed a hand search of the International Journal of Integrated Care and the Journal of Integrated Care.

The inclusion criteria included: 1) studies that assessed strategies explicitly aiming to integrate care for pregnant women and newborns. They could aim exclusively at integrating care for pregnant women and newborns, or they could be part of a population-based strategy for integrating care that explicitly included pregnant women and newborns. We considered all types of integration strategies (systemic, functional, service/professional, clinical, normative, and organisational integration strategies) and their combinations. We also included 2) studies that assessed integration strategies implemented for maternal care in low- or middle–income countries as defined by the World Bank country classification [38], 3) studies that assessed whether care had been integrated effectively, evaluating changes in the coordination of the care process, patient-focused organisation of care, communication with patients and family, collaboration with primary care and follow-up to the care process, and interprofessional and inter-organisational collaboration. Changes in these features of the organisation of care could be assessed using qualitative or quantitative methods. 4) Only studies in English and Spanish published during the last 5 years were considered for inclusion. We considered Spanish publications because many Latin American countries publish research in this language, and they are all low- and middle-income countries. Qualitative studies and quantitative studies based on primary data collection and secondary data analysis were included.

When appropriate, usual care, fragmented care, or care before integration strategies were implemented were used as comparators.

The exclusion criteria included: 1) Systematic and scoping reviews, editorials, and narrative reviews of the literature were excluded, although we checked their references for relevant publications. 2) Studies that did not have an abstract were excluded, as we used the abstract to screen publications. 3) We excluded studies published only as conference abstracts because it wasn’t possible to perform an adequate quality assessment of this form of publication. 4) We excluded studies that assessed only health outcomes (morbidity and mortality), as the scope of this article is on the organisational outcomes and not the health outcomes.

The references and abstracts of all the identified publications were downloaded or manually entered into Mendeley® to identify duplicates. After duplicates were eliminated, title and abstract screening were conducted independently by two reviewers (LvdW, LP, DSS), following the exclusion and eligibility criteria to eliminate non-relevant publications. Next, the full text of the remaining publications was assessed by one reviewer and verified by a second reviewer (after initial assessment by LvdW, the second reviewer, LP, also assessed the full text of the documents, and reviewed the judgement of the first reviewer). Disagreements were limited and resolved without difficulty through discussion. We documented the study selection and screening process in a flow diagram following the Preferred Reporting Items for Systematic Reviews and Meta-Analyses Statement [32]. We designed a data extraction form to extract information for each type of integration strategy (systemic integration, functional integration, service integration, clinical integration, normative integration, organisational integration) and the outcomes assessed for each strategy.

We critically appraised the included studies using an adapted quality appraisal tool applicable to each type of study (Qualitative studies: CASP questionnaire, and Quality assessment tool for quantitative studies [39,40,41], and the case study critical appraisal tool for case studies [42]). We constructed common categories to grade the quality of each quality appraisal tool (high, good, moderate, low, poor) and classified the overall quality of each of the publications according to these categories. We used the quality appraisal results to identify gaps in the literature caused by poor quality research, but not to exclude publications from the review.

## Results

**Figure 1:**
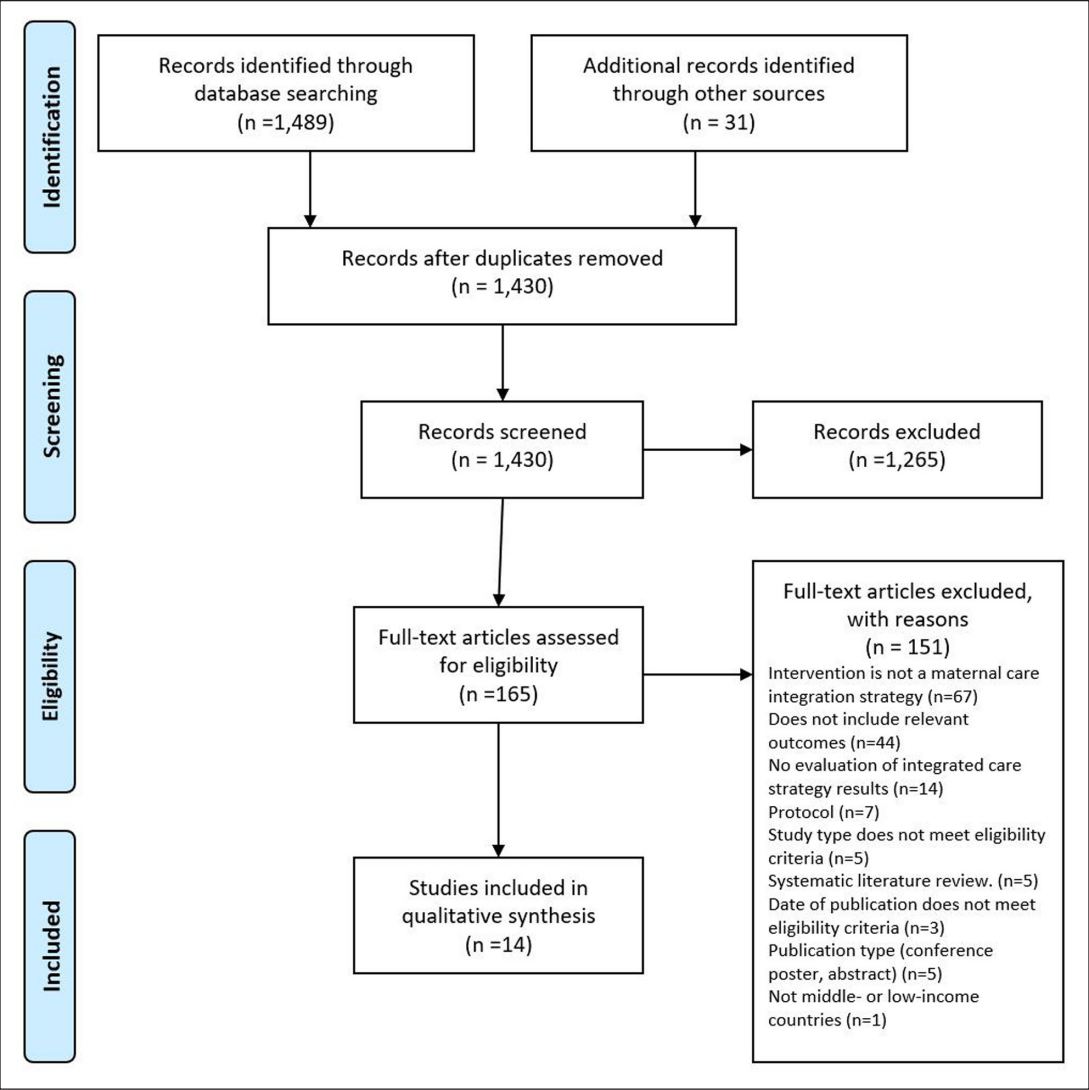
PRISMA diagram.

Figure 1 presents an overview of the study selection process. We identified a total of 1,489 records through the electronic database search and thirty-one records through other sources (snowballing, Google Scholar, and hand search of Integrated Care Journals), resulting in a total of 1,520 publications. After duplicate removal, 1,430 articles remained. Following title and abstract screening, we assessed 165 publications for eligibility through full-text review. Of these 165 articles, we excluded 151: sixty-seven publications were excluded because they did not assess maternal care integration strategies, 44 because they did not include relevant outcomes, 14 because they did not evaluate the results achieved by the care integration strategies, seven publications because they were research protocols, five publications because the study type did not meet the eligibility criteria, three studies because their date of publication did not meet the eligibility criteria, five because of publication type (conference abstract), and one publication because the study was not from a low- or middle-income country. We assessed the publications included in five systematic reviews for eligibility, but we ultimately excluded them because the primary studies they included did not meet the eligibility criteria. Appendix 2 contains a list of the reasons for excluding each publication. Finally, we included 14 publications that met the eligibility criteria [43,44,53,54,55,56,45,46,47,48,49,50,51,52]. The characteristics of the included studies are summarised in Appendix 3.

We included four quantitative studies. Two of them were considered high-quality studies [50,53]; one was of good quality [54], and one was of moderate quality [48]. We included three mixed-method studies. After critical appraisal, we considered two studies to be high-quality studies [50,53]. One of the studies was of moderate quality [56]. We included four qualitative studies. Three of them were high-quality studies [43,47,49], and one was of moderate quality [46]. Finally, we included three case studies. Two of them had good methodological quality [45,51], and one was of moderate quality [44]. The detailed formats for the quality appraisal can be found in Appendix 4.

### Organisational integration strategies

Four publications [43,44,45,46] described the outcomes of strategies that aimed to improve the coordination of maternal care through constructing new inter-organisational relationships. Table 3 presents a summary of these strategies. All the strategies aimed to establish links between organisations providing health care, but they differed in the methods used to link the organisations and the strength of the links formed between them. These strategies had effects on inter-organisational collaboration and coordination of care.

**Table 3:** Organisational integration strategies.


REFERENCE	COUNTRY	NAME	STRATEGY DESCRIPTION	OUTCOME

Dillip et al. [43]	Tanzania	Training in Kibaha district	Joint training to link health workers	Increased inter-organisational collaboration Increased collaboration with primary care

Kalita et al. [44]	India	Integrating health and nutrition programs in Chhattisgarh, India	Merger between health programmes	Improved coordination of care

Kearns et al. [45]	Bangladesh	The Manoshi Project	Building a direct referral system	Increased collaboration with primary care Increased inter-organisational collaboration

	Kenya	Jacaranda Health	Assessment of availability and capabilities of facilities for referral	Increased collaboration with primary care Increased inter-organisational collaboration

Lhamsuren et al. [46]	Mongolia	Reaching Every District	Partnership through referrals from one organisation to another	Increased inter-organisational collaboration


Lhamsuren et al. [46] and Dillip et al. [43] described strategies that aimed to create links between organisations through alliances established at the level of employees, who were put in charge of referral from one organisation to another. Lhamsuren et al. [46] described the “Reaching Every District” strategy in Mongolia to coordinate civil registration services and access to needed health services through a new partnership in which clients from health services were referred to the civil registration office and vice versa.

Dillip et al. [43] described a strategy that consisted of joint training to formalise the links between community health workers, accredited drug dispensers, and health facility staff in the Kibaha district in Tanzania.

Kearns et al. [45] described two strategies in which one of the core components was establishing referral networks. In Nairobi, during the early planning of the Jacaranda health centre, the management team assessed the capacity of higher-level facilities to admit their complicated labour and delivery cases and perform caesarean sections when needed. In Bangladesh, the Manoshi project created a network of urban community health workers who provide basic primary care. They secure access to emergency care through partnerships with referral facilities.

Kalita et al. [44] described an organisational integration strategy that included characteristics of normative integration strategy. This strategy aimed to address the gaps in delivery of services and in human resource abilities and performance by horizontal and vertical integration between the child and maternal health and nutrition program structures. A state-level institution was created to lead the integration process and conceptualise and operationalise all innovations at the decentralised levels (i.e., create a common frame of reference). Furthermore, the strategy included joint training sessions for health and nutrition programme workers.

### Service/professional integration strategies

Four publications [45,47,48,49] discussed strategies for integrating maternal care by creating new interactions between health professionals. These strategies, which are listed in Table 4, impacted inter-organisational collaboration, follow-up of the care process, and the patient-centeredness of care.

**Table 4:** Service/professional integration strategies.


REFERENCE	COUNTRY	NAME	STRATEGY DESCRIPTION	OUTCOME

Jiang et al. [47]	China	Safe motherhood program in rural Guangxi Zhuang Autonomous	Traditional birth attendants as liaisons	Improvement of follow-up

Kearns et al. [45]	Ethiopia	Health extension worker (HEW) programme	Community health workers as liaisons	Increased collaboration with primary care

	Pakistan	Lady Health Worker	Community health workers as liaisons	Increased collaboration with primary care

Mwaniki et al. [48]	Kenya	The Kenya Kwale district improvement collaborative	Traditional birth attendants as liaisons	Increased collaboration with primary care Improvement of follow-up

Orya et al. [49]	Somalia and Sierra Leone	Improving the reproductive and sexual health of internally displaced people, Maroodi Jeex, Somaliland. Building capacity for the improvement of infant and maternal health in northern Bombali, Sierra Leone	Traditional birth attendants as liaisons	Improvement of follow-up. Making care more patient-centred


Jiang et al., [47] Orya et al., [49] Mwaniki et al., [48] and Kearns et al. [45] described strategies that consisted of creating a new role – liaison to the primary healthcare system. These strategies were implemented in China, Somalia, Sierra Leone, Kenya, Pakistan, and Ethiopia. They differed in that the strategies described by Jiang et al., [47] Orya et al., [49] and Mwaniki et al. [48] worked to redefine the role of former traditional birth attendants to act as liaisons, to decrease non-skilled birth attendance. On the other hand, in the strategies described by Kearns et al., [45] community health workers were the ones who acted as liaisons with the primary healthcare system. In both cases, there were increases in the proportion of women who received comprehensive antenatal care and other maternal care services; there was an increase in the demand for maternal and newborn services, and in the follow-up of the care process, and the acceptability of the care improved.

### Clinical integration strategies

Five publications [50,52,53,54] included three different strategies for coordinating care through understanding it as a single process. All of these clinical integration strategies were combined with functional integration strategies. Table 5 details the identified clinical integration strategies. These strategies consisted of decision support tools and clinical practice guidelines embedded in systems for information sharing between healthcare providers. They influenced the follow-up of the care process, coordination of the care process, and interprofessional collaboration.

**Table 5:** Clinical integration strategies.


REFERENCE	COUNTRY	NAME	STRATEGY DESCRIPTION	OUTCOME

Carmichael et al. [50] Balakrishnan et al. [51] Borkum et al. [52]	India	Information communication Technology Continuum of Care Service	Mobile technology tool for frontline workers	Improved coordination of the care process Improved interprofessional collaboration Improved follow-up of the care process

Graven et al. [53]	Belize	Belize Integrated Patient-centred Country Wide Health Information System (BHIS)	Country-wide health information system with an embedded maternal care program management algorithm	Improved follow-up of care

Osaki et al. [54]	Indonesia	Maternal and child health handbook	Home-based record	Improved follow-up of the care process


Osaki et al. (44) described a home-based maternal care record that includes information about the services that should be part of maternal care. Different health providers use this shared record to register information about the care process and coordinate the delivery of care. The authors found that a higher proportion of mothers who possessed home-based maternal care records received continuous maternal care than did women who did not have a record (65.7% vs 42.3%).

Carmichael et al., [50] Balakrishnan et al., [51] and Borkum et al. [52] described and assessed a strategy for maternal care integration implemented in Bihar (India). It consisted of a mobile technology tool designed for community-based frontline health workers. It integrated multiple functions to assist frontline health workers in their duties, including registration and tracking of beneficiaries, automated scheduling of home visits, provision of health information through videos, guiding protocols for conducting home visits through checklists, a feature to track child immunisations, and supervisory tools.

Graven et al. [53] also reported a strategy that consisted of a countrywide health information system with an embedded maternal care program management algorithm. Within one year of the system’s deployment, the follow-up of the care process improved. Over 90% of all healthcare encounters were entered into the system, and after two years, this increased to over 95%.

### Functional integration strategies

Two publications [55,56] described functional integration strategies that were not combined with clinical integration strategies. They affected inter-organisational collaboration, the follow-up of the care process, patient-centeredness of care, and communication with patients and family (Table 6).

**Table 6:** Functional integration strategies.


REFERENCE	COUNTRY	NAME	STRATEGY DESCRIPTION	OUTCOME

Jalloh-Vos et al. [55]	Sierra Leone	Mobile communication strategies	Establishing a virtual private network (VPN) and distributing mobile phones, SIM cards, and prepaid phone credit	Improved communication with patients and family Increased interprofessional collaboration Increased collaboration with primary care

Meyer et al. [56]	South Africa	AitaHealth ™	Home-based and facility-based healthcare mobile information system	Improved follow-up of the care process


Meyer et al. [56] described a strategy in which home-based and facility-based healthcare information was linked using a smartphone-based platform (AitaHealth™). According to the authors, the availability of health information for community-oriented primary health workers improved patient traceability and follow-up at home, making timely referrals possible. However, wrong addresses or personal identifications posed a challenge for tracing pregnant women.

Jalloh-Vos et al. [55] described a functional integration strategy through mobile communications strategies. The strategy included establishing a virtual private network, distributing mobile phones and SIM cards to peripheral health units, and prepaid phone credit.

We did not identify publications that described the organisational outcomes of normative or systemic integration strategies in the context of maternal health care. Table 7 summarises the types of maternal care strategies implemented in low- and middle-income countries and their effects on the organisation of care.

**Table 7:** Effect of each type of maternal care integration strategy on organisational outcomes.


TYPE OF INTEGRATION STRATEGY	OUTCOME					

	INTER- ORGANISATIONAL COLLABORATION	FOLLOW-UP OF THE CARE PROCESS	PATIENT-CENTRED CARE	COMMUNICATION WITH PATIENTS AND FAMILY	COORDINATION OF CARE PROCESS	INTER- PROFESSIONAL COLLABORATION

**Organisational**	**+**	**NI**	**NI**	**NI**	**+**	**NI**

**Service/ professional**	**+**	**+**	**+**	**NI**	**NI**	**NI**

**Functional**	**+**	**+**	**+**	**+**	**NI**	**NI**

**Clinical plus functional**	**NI**	**+**	**NI**	**NI**	**+**	**+**


+: type of strategy listed in the corresponding line influenced the organisational outcome listed in the column. NI: effects of type of strategy listed on the line not identified for the type of outcome listed in the corresponding column.

## Discussion

This study aimed to identify and describe the strategies that have been used in low -and middle-income countries to integrate maternal care (i.e., deliver coherent, coordinated, and continuous services by cooperating actors across the care continuum) and their effect on the organisation of care.

We identified five types of strategies that have been implemented in low- and middle-income countries. 1) Organisational integration strategies and 2) organisational combined with normative strategies led to an increased inter-organisational collaboration through the formation of links – with different degrees of strength - between organisations. These strategies were also associated with changes in the coordination of care. 3) Service/professional integration strategies, which consisted mainly of liaisons who acted as a link between the healthcare system and patients, increased the collaboration between organisations and seemed to improve the ability of health care to respond to variances, risks of complications, and outcomes of maternal care (i.e., follow-up of the care process). 4) Functional integration strategies had effects on multiple organisational outcomes, including the inter-organisational collaboration, follow-up, and patient-centeredness of the care process. 5) Clinical integration strategies coupled with functional integration strategies – such as medical records and mobile technology – were used to simplify access to information supporting decision-making for health professionals. In general, these strategies aimed to provide guidance and support for decision-making during care. This combination of strategies influenced the follow-up of the care process, the coordination of care, and interprofessional collaboration.

Overall, most types of strategies influenced inter-organisational collaboration and the follow-up of the care process. Some strategies also affected the patient-centeredness of care, the coordination of the care process, communication with patients and family, and interprofessional collaboration. Our findings suggest that different types of strategies may lead to comparable organisational outcomes (e.g., organisational integration strategies and professional or service integration strategies may similarly influence inter-organisational collaboration). This finding needs further research, as some types of strategy may be easier to implement in a resource-constrained context – and thus preferred if they lead to comparable outcomes.

Strategies that impact inter-organisational collaboration may be widespread because this characteristic of care organisation may be perceived as particularly important for reducing maternal and neonatal deaths. For instance, inter-organisational collaboration may be crucial for a successful referral for delivery by a skilled health provider, which is considered the most important intervention to reduce maternal and neonatal mortality [57]. It may also be necessary for optimising the speed at which a referral toward emergency obstetric care may be achieved. Timely referral to emergency obstetric care is critical for reducing maternal mortality [58]. Because of this, inter-organisational collaboration may be particularly important in the context of maternal care integration.

An important finding was that most types of strategies described in theory (i.e., organisational, service/professional, functional, and clinical strategies) have been implemented and studied in low- and middle-income countries. The most frequent types of strategies were organisational and service/professional integration strategies. We did not identify any publications describing systemic integration strategies implemented in low- and middle-income countries.

Organisational strategies aimed to establish links between independent organisations, but they differed in the methods used to link the organisations and the strength of the links formed between them. While in some cases organisations were fully merged, in other strategies, organisations formed weaker links between each other, such as referral networks or joint training. However, they all affected inter-organisational collaboration and collaboration with primary care. This finding supports evidence from previous observations of the effect of organisational alliances (without full integration) on inter-organisational collaboration [59]. However, further research is necessary to establish whether the strength of organisational links influences the degree of inter-organisational collaboration achieved.

The establishment of liaisons that act as a formal link between the health system and patients was one of the most frequently used integration strategies implemented in low- and middle-income countries. A systematic literature review that gathered evidence about maternal care coordination programmes implemented in the United States found that a similar strategy, individual case management, was one of their most frequent components. It consisted of visits by a case manager, regular contact between patient and case manager to discuss pregnancy questions and concerns, and the case manager initiating and scheduling appointments with other specialists or programs. However, in the United States, case managers were usually nurses or general practitioners, while in low- and middle-income countries, liaisons were generally community health workers and traditional birth attendants [27].

Our study found that community health workers as liaisons made care more focused on a patient’s needs and preferences. This finding is consistent with recent research that identified that community healthcare workers frequently act as mediators between individuals and health services, acting as cultural mediators, serving as patient and community advocates, assisting patients with appropriate health services utilisation, providing psychosocial support to patients, providing health education and motivation for behaviour change to community members [60]. Although budget constraints are the likely reason behind community health workers and traditional birth attendants acting as liaisons in low- and middle-income countries instead of nurses and general practitioners, the former may offer an advantage in some contexts because of their practical knowledge of the community and its culture.

Kroll-Desrosiers et al. [27] found that having a team approach to decision-making was a frequent component of maternal care coordination programmes implemented in the United States. In contrast, we did not identify any publication describing this type of service/professional integration strategy in low- and middle-income countries. The absence of this type of integration strategy may be related to differences in the number of health workers and professional backgrounds involved in the maternal care continuum in low- and middle-income countries [61].

Most of the publications reported on strategies for maternal care integration in countries in Asia and Africa. Only one publication was from Latin America and the Caribbean (Belize). Although several care integration strategies may have been implemented in other low- and middle-income countries, these may be unstudied or unpublished.

The findings of this systematic literature review may be somewhat limited by the fact that in the included publications, organisational outcomes had not been defined explicitly according to the pre-specified categories (coordination of the care process, patient-focused organisation of care, communication with patients and family, inter-organisational collaboration and collaboration with primary care, follow-up of the care process and interprofessional collaboration). Because of this, it was necessary to categorise outcomes that were considered equivalent to the pre-specified organisation outcomes (i.e., we established whether the concept behind the outcomes assessed by each study matched the pre-established organisational outcomes and categorised the assessed outcomes accordingly). Our categorisation may lead to differences in the interpretation of the outcomes and limited reproducibility.

Although instruments for measuring the integration of care have been developed [23,24], they have not been validated in low- and middle-income countries and, to our knowledge, they have not been used for measuring the effective integration of maternal care. Future studies that validate the operationalisation of organisational outcomes in low- and middle-income countries are needed to understand the comparative effectiveness of care integration strategies on maternal care.

Care integration strategies can be part of complex interventions that include several components. Integration strategies may be coupled with interventions that strengthen the health system or they may be part of a broader strategy for improving the quality of health services. These interventions may act independently and interdependently. Because of this, some of the included interventions may have included additional elements that influenced the organisation of care. However, we categorised each strategy according to its main components and linked each of them to the specific organisational outcomes that were influenced by them. Thus, a better understanding of the effects that each type of strategy may have on the organisation of care provides insights for the design of combined integration strategies that act upon issues identified in the organisation of care.

Another source of uncertainty that must be considered in interpreting the findings of this systematic review is that because care integration strategies are complex, designing randomised controlled studies to evaluate them is burdensome [62]. Accordingly, research into these strategies has been limited. Furthermore, some of the studies were of moderate methodological quality [44,46,48,56]. Therefore, the available evidence is subject to relevant sources of bias.

Although other methodologies, such as the integrative review might allow a better combination of the findings from the diverse types of research methodologies included [63], we chose to conduct a systematic literature review as this type of secondary research is considered the most reliable source of evidence to guide practice [64]. This type of review is useful for identifying what has been written on a subject, reveals interpretable patterns, allows identification of areas where further research is required, and can be the basis for theory and framework development [65]. Furthermore, it can include all types of primary research, regardless of their methodology [64].

Notwithstanding these limitations, this systematic review provides an overview of the diverse strategies that have been used in low- and middle-income countries to integrate maternal care, as well as their effects on the organisation of maternal care. The review allows a better understanding of strategies used in contexts where resources are significantly constrained and where, in addition to integrating maternal care, there is a need to strengthen the health system in general. These insights can guide the development of policies that enable the implementation of successful maternal care integration strategies.

## Conclusions

Most types of the strategies described in theory have been implemented in low- and middle-income countries. Overall, most types of strategies influenced inter-organisational collaboration and the follow-up of the care process. Some strategies also affected the patient-centeredness of care, the coordination of the care process, communication with patients and family, and interprofessional collaboration. Our findings suggest that different types of strategies may lead to comparable organisational outcomes – e.g., organisational integration strategies and professional or service integration strategies may similarly influence inter-organisational collaboration. The latter may play a critical role in the context of maternal care integration, as it aligns with changes in the organisation of care that are considered necessary for reducing maternal and neonatal mortality.

## Additional File

The additional file for this article can be found as follows:

10.5334/ijic.6254.s1 Appendices. Appendix 1 to 4.
