# Supplementary material for: Integrated Maternal Care Strategies in Low- and Middle-Income Countries: A Systematic Review
Source: Int J Integr Care. 2022 Jun 22;22(2):26. doi: 10.5334/ijic.6254 (PMC9231572; doi:10.5334/ijic.6254)
Supplement: Appendices. — Appendix 1 to 4. [file ijic-22-2-6254-s1.pdf]

Appendices:

Appendix 1 Search strategies

Search strategy	
**Database**	MEDLINE
**Search engine**	OVID
**Date**	04-11-2019
**Publication date restrictions**	Last 5 years
**Language restrictions**	None
**Other limits**	None
**Search strategy**	1.exp Mothers/ (41275)<br>2.exp Maternal Health/ (1143)<br>3.exp Prenatal Care/ (26389)<br>4.exp Prenatal Education/ (215)<br>5.exp Maternal Welfare/ (6532)<br>6.Pregnancy/ (855968)<br>7.Pregnancy Complications/ (88717)<br>8.exp Breast Feeding/ (36439)<br>9.exp Midwifery/ (18749)<br>10.exp Maternal-Child Health Centers/ (2304)<br>11.Maternal Health Services/ (13344)<br>12.exp Maternal-Child Health Services/ (645)<br>13.exp Perinatal Care/ (9600)<br>14.exp Postnatal Care/ (5377)<br>15.exp Infant, Newborn/(592933)<br>16.exp Neonatal Screening/(9791)<br>17.Prenatal.ti. (35110)<br>18.Pre-natal.ti. (224)<br>19.Pregnant.ti. (37986)<br>20.Pregnanc*.ti. (164740)<br>21.Maternity.ti. (5519)<br>22.Maternal.ti. (67801)<br>23.Mother*.ti. (40190)<br>24.Childbirth.ab,ti. (14264)

	25.child birth.ab,ti.(630)<br>26.obstetric care.ti. (743)<br>27.antenatal*.ti. (8727)<br>28.neonatal*.ti. (74373)<br>29.perinatal*.ti. (22437)<br>30.postnatal*.ti. (21001)<br>31.post-natal*.ti. (1256)<br>32.postpartum.ti. (15350)<br>33.post-partum.ti. (2484)<br>34.preterm.ti. (27696)<br>35.birth outcome*.ti. (1432)<br>36.newborn.ti. (53302)<br>37.Midwife-led.ti. (122)<br>38.birth attendant*.ti. (403)<br>39.Breastfeed*.ti. (8861)<br>40.breast feed*.ti. (4881)<br>41.1 or 2 or 3 or 4 or 5 or 6 or 7 or 8 or 9 or 10 or 11 or 12 or 13 or 14 or 15 or 16 or 17 or 18 or 19 or 20 or 21 or 22 or 23 or 24 or 25 or 26 or 27 or 28 or 29 or 30 or 31 or 32 or 33 or 34 or 35 or 36 or 37 or 38 or 39 or 40(1412078)<br>42.Integrated care.tw. (2922)<br>43.integrat*.jw. (32418)<br>44.(care or healthcare).jw. (342083)<br>45.43 and 44(159)<br>46.(Integrat* or coordinat*) .ab,ti.(553929)<br>47.((Integrat* or coordinat*) and (care and health)) .ab,ti.(42010)<br>48.(manag* or systems or model* or organisat* or organizati* or service delivery).ab,ti.(3934298)<br>49.(Disease management or Case management).ab,ti.(20638)<br>50.(Organisational integration or Vertical integration or horizontal integration or Organisational objectives or Organisational policy or Organizational Case Studies or interinstitutional relations or Community-institutional relations or Cooperative behavi* or (Organization and Administration) or Health services administration or Hospital restructuring or Multi-institutional systems or (Efficiency adj2 Organi*ational) or restructur* or re-structur* or affiliat* or

	partner* or (alliance* and institut*) or collaborat* or network* or interinstitutional or inter-institutional).ab,ti.(613103)<br>51.((Integration and (functional or support or "back-office" or "electronic medical records" or "information technology")) or functions).ab,ti.(534187)<br>52.(Patient care team or Intersectoral collaboration or team* or teamwork* or interprofessional relations or interdisciplinary communication or attitude of health personnel or multidisciplinary or multi-disciplinary or interdisciplinary or inter-disciplinary or transdisciplinary or trans-disciplinary or collaborati* or collaborative practice or ((Social accountability or accountab* or responsib*) and (shared or joint*)) or ((Service or professional) and integration)).ab,ti.(302539)<br>53.(Clinical integration strategies or clinical practice guideline* or Pathway* or Continuity of patient care or case manage* or critical pathways or care plan* or Patient care planning or interdisciplinary communication or pathway or community matron* or (Clinical integration strategies or clinical practice guideline* or Pathway* or Continuity of patient care or case manage* or critical pathways or care plan* or Patient care planning or interdisciplinary communication or pathway or community matron*) or ((Care or service*) and (navigation or continuum or transition*OR fragment* or continuous or continuity or discontinuity or coordinat* or co-ordinat* or linkage* or seamless*))).ab,ti.(1011282)<br>54.(Normative integration or organi*ational culture or organi*ational development or culture change or professional culture or professional development or professional values or (shared and (mission or vision or values or culture or organisational culture or social values or social norms))).ab,ti.(18282)<br>55.(((coherent or consistent) and (rules or policies or regulations or directions)) or systemic integration).ab, ti (8419)<br>56.50 or 51 or 52 or 53 or 54 or 55(2167155)<br>57.46 and 48(211899)<br>58.42 or 45 or 47 or 49 or 57(245485)<br>59.(middle income econom* or middle income econom*OR Low income countr* or Middle income countr*OR developing countries

	or less developed countries or third-world countries or under-developed countries or poor countries or less developed countries or under developed countries or less developed nations or third world nations or under developed nations or developing nations or poor nations or poor economies or third world economies or developing economies or under developed economies or less developed economies).ab,ti.(6079)<br>60.(Afghanistan or Guinea or Rwanda or Benin or Guinea ad2 Bissau or Senegal or Burkina Faso or Haiti or Sierra Leone or Burundi or Korea or Somalia or Central African Republic or Liberia or South Sudan or Chad or Madagascar or Tanzania or Comoros or Malawi or Togo or Congo or Mali or Uganda or Eritrea or Mozambique or Zimbabwe or Ethiopia or Nepal or Gambia or Niger or Armenia or Kiribati or Solomon Islands or Bangladesh or Kosovo or Sri Lanka or Bhutan or Kyrgyz Republic or Sudan or Bolivia or Lao PDR or Swaziland or Cabo Verde or Lesotho or Syria* or Cambodia or Mauritania or Tajikistan or Cameroon or Micronesia or Timor or Congo or Moldova or Tonga or (C*te and Ivoire) or Mongolia or Tunisia or Djibouti or Morocco or Ukraine or Egypt or Myanmar or Uzbekistan or El Salvador or Nicaragua or Vanuatu or Ghana or Nigeria or Vietnam or Guatemala or Pakistan or (West Bank and Gaza) or Honduras or Papua New Guinea or Yemen or India or Philippines or Zambia or Indonesia or Samoa or Kenya or (S*o Tom* adj2 Principe) or Albania or Ecuador or Montenegro or Algeria or Fiji or Namibia or American Samoa or Gabon or Palau or Angola or Georgia or Panama or Argentina or Grenada or Paraguay or Azerbaijan or Guyana or Peru or Belarus or Iran or Romania or Belize or Iraq or Russia* or (Bosnia or Herzegovina) or Jamaica or Serbia or Botswana or Jordan or South Africa or Brazil or Kazakhstan or s*t Lucia or Bulgaria or Lebanon or S*t Vincent or Grenadines or China or Libya or Suriname or Colombia or Macedonia or Thailand or Costa Rica or Malaysia or Turkey or Cuba or Maldives or Turkmenistan or Dominica or Marshall Islands or Tuvalu or Dominican Republic or Mauritius or Venezuela or Equatorial or Guinea).ab,ti.(842196)<br>61.59 or 60 (846381)

	62.41 and 58 and 61 and 56 (1180)<br>63.limit 62 to (medline and last 10 years) (793)<br>64.limit 63 to (medline and last 5 years) (549)
**Records retrieved**	549

**Search strategy**	
**Database**	Healthcare administration database
**Search engine**	ProQuest
**Date**	04-11-2019
**Publication date restrictions**	Last 5 years
**Language restrictions**	None
**Other limits**	Peer reviewed journals
**Search strategy**	ab((maternal child health centers) OR (postnatal care) OR (perinatal care) OR (prenatal care) OR (antenatal$)) AND ab((Integrated care OR integrated care OR Integrat$ OR coordinat$ OR services OR delivery OR management OR systems OR model OR organi$ational OR Disease management OR Case management)) AND ab((middle income econom$ OR middle income econom$ OR Low income countr$ OR Middle income countr$ OR developing countries OR less developed countries OR third-world countries OR underdeveloped countries OR poor countries OR less developed countries OR under developed countries OR less developed nations OR third world nations OR under developed nations OR developing nations OR poor nations OR poor economies OR third world economies OR developing economies or under developed economies or less developed economies))
**Records retrieved**	52

**Search strategy**	
**Database**	Science Citation Index (SCI), Social Sciences Citation Index (SSCI), Arts & Humanities Citation Index (A&HCI)

**Search engine**	Web of Science
**Date**	04-11-2019
**Publication date restrictions**	Las 5 years
**Language restrictions**	none
**Other limits**	none
**Search strategy**	ALL FIELDS: ((((maternal or prenatal or antenatal or perinatal or postpartum or postnatal) AND care) AND ("integrated care" or "coordinated care") AND ((middle income countries) or (low income countries))))
**Records retrieved**	4

**Search strategy**	
**Database**	
**Search engine**	SCOPUS
**Date**	04-11-2019
**Publication date restrictions**	None
**Language restrictions**	None
**Other limits**	None
**Search strategy**	(TITLE-ABS-KEY (((maternal OR prenatal OR antenatal OR perinatal OR postnatal OR postpartum) AND (care OR services OR healthcare))) AND TITLE-ABS-KEY ((((integrat$ OR coordinat$ OR continu$ OR seamless OR patient-cent$ed OR disease AND management OR case AND management OR shared OR transitional) AND care) OR integrated AND delivery AND systems)) AND TITLE-ABS-KEY (((middle AND income OR low AND income OR middle-income OR low-income OR underdeveloped OR under AND developed OR developing OR poor OR less AND developed OR third AND world OR third-world) AND (countries OR nations OR econom$))))
**Records retrieved**	4

Search strategy	
**Database**	EMBASE
**Search engine**	Elsevier
**Date**	04-11-2019
**Publication date restrictions**	Last 5 years
**Language restrictions**	none
**Other limits**	Not medline
**Search strategy**	1. ('maternal health':ab,ti,kw(7622)<br>2. ('prenatal care':ab,ti,kw(11835)<br>3. ('maternal welfare'/exp(14699)<br>4. ('midwifery':ab,ti,kw(10773)<br>5. ('maternal-child health centers'/exp(2185)<br>6. ('maternal health services':ab,ti(746)<br>7. ('maternal-child health services'/exp(2185)<br>8. ('newborn screening':ab,ti(8737)<br>9. (prenatal:ti(46668)<br>10. ('obstetric care':ab,ti(4018)<br>11. (antenatal$:ti(12351)<br>12. (neonatal$:ti(99071)<br>13. (perinatal$:ti(30332)<br>14. (postnatal$:ti(26496)<br>15. (postpartum:ti(22161)<br>16. (birth attendan$":ab(ti")<br>17. (#2 OR #4 OR #5 OR #6 OR #7 OR #13 OR #14 OR #15 OR #8 OR #9 OR #12 OR #11 OR #10(298444)<br>18. (integrat$:ab,ti(61553)<br>19. (coordinat$:ab,ti(47382)<br>20. (continu$:ab,ti(178243)<br>21. (seamless:ab,ti(4674)<br>22. (patient cent$ed":ab(ti")<br>23. (shared:ab,ti(154849)<br>24. (transitional:ab,ti(38824)<br>25. (#18 OR #19 OR #20 OR #21 OR #22 OR #23 OR #24(504351)

	26. (care OR service$ OR model$ OR healthcare OR delivery OR 'health care delivery':ab,ti,kw(9880286)<br>27. (#25 AND #26(240856)<br>28. ('organizational integration':ab,ti(56)<br>29. ('vertical integration':ab,ti(552)<br>30. ('horizontal integration':ab,ti(200)<br>31. ('organizational objectives':ab,ti,kw(94)<br>32. ('organizational policy' OR 'organizational case studies' OR 'interinstitutional relations' OR 'institutional relations':ab,ti(1286)<br>33. ("cooperative behave$" OR 'organisation and administration' OR 'health services administration':ab,ti(1241)<br>34. ('health services administration' OR 'hospital restructuring' OR 'multi institutional systems' OR 'organizational efficacy' OR network$ OR 'interinstitutional' OR 'inter institutional':ab,ti(716965)<br>35. (#28 OR #29 OR #30 OR #31 OR #32 OR #33 OR #34(719526)<br>36. (shared AND (mission:ab,ti,kw OR vision:ab,ti,kw OR values:ab,ti,kw OR culture:ab,ti,kw)(13008)<br>37. ('organi$ational culture':ab,ti(2232)<br>38. ('social values':ab,ti,kw(1009)<br>39. ('social norms':ab,ti,kw(4310)<br>40. ('normative integration':ab,ti(12)<br>41. (#37 AND #38 OR #39(4312)<br>42. (shared(160536)<br>43. (#41 AND #42(137)<br>44. (#36 OR #43(13121)<br>45. ("clinical practice guideline$" OR "pathway$" OR 'continuity of patient care' OR "case manage$" OR 'critical pathways' OR "care plan$" OR 'patient care planning' OR 'interdisciplinary communication' OR "community matron$" OR 'clinical integration strategies' OR 'care navigation':ab,ti,kw(36417687)<br>46. (#35 OR #44 OR #45(36417687)<br>47. ('middle income' OR 'low income' OR underdeveloped OR 'under developed' OR developing OR poor OR 'less developed' OR 'third world':ab,ti,kw(1518114)<br>48. (countries OR nations OR econom$:ab,ti,kw(481192)

	49. (#47 AND #48(143500)<br>50. ('afghanistan' OR 'guinea' OR 'rwanda' OR 'benin' OR 'guinea bissau' OR 'senegal' OR 'burkina faso' OR 'haiti' OR 'sierra leone' OR 'burundi' OR 'korea' OR 'somalia' OR 'central african republic' OR 'liberia' OR 'south sudan' OR 'chad' OR 'madagascar' OR 'tanzania' OR 'comoros' OR 'malawi' OR 'togo' OR 'mali' OR 'uganda' OR 'eritrea' OR 'mozambique' OR 'zimbabwe' OR 'ethiopia' OR 'nepal' OR 'gambia' OR 'niger' OR 'armenia' OR 'kiribati' OR 'solomon islands' OR 'bangladesh' OR 'kosovo' OR 'sri lanka' OR 'bhutan' OR 'kyrgyz republic' OR 'sudan' OR 'bolivia' OR 'lao pdr' OR 'swaziland' OR 'cabo verde' OR 'lesotho' OR 'syria$' OR 'cambodia' OR 'mauritania' OR 'tajikistan' OR 'cameroon' OR 'micronesia' OR 'timor' OR 'congo' OR 'moldova' OR 'tonga' OR 'ivoire' OR 'mongolia' OR 'tunisia' OR 'djibouti' OR 'morocco' OR 'ukraine' OR 'egypt' OR 'myanmar' OR 'uzbekistan' OR 'el salvador' OR 'nicaragua' OR 'vanuatu' OR 'ghana' OR 'nigeria' OR 'vietnam' OR 'guatemala' OR 'pakistan' OR 'gaza' OR 'honduras' OR 'papua new guinea' OR 'yemen' OR 'india' OR 'philippines' OR 'zambia' OR 'indonesia' OR 'samoa' OR 'kenya' OR 's$o tom$ ' OR 'albania' OR 'ecuador' OR 'montenegro' OR 'algeria' OR 'fiji' OR 'namibia' OR 'american samoa' OR 'gabon' OR 'palau' OR 'angola' OR 'georgia' OR 'panama' OR 'argentina' OR 'grenada' OR 'paraguay' OR 'azerbaijan' OR 'guyana' OR 'peru' OR 'belarus' OR 'iran' OR 'romania' OR 'belize' OR 'iraq' OR 'russia$' OR 'bosnia or herzegovina' OR 'jamaica' OR 'serbia' OR 'botswana' OR 'jordan' OR 'south africa' OR 'brazil' OR 'kazakhstan' OR 's$t lucia' OR 'bulgaria' OR 'lebanon' OR 's$t vincent' OR 'grenadines' OR 'china' OR 'libya' OR 'suriname' OR 'colombia' OR 'macedonia' OR 'thailand' OR 'costa rica' OR 'malaysia' OR 'turkey' OR 'cuba' OR 'maldives' OR 'turkmenistan' OR 'dominica' OR 'marshall islands' OR 'tuvalu' OR 'dominican republic' OR 'mauritius' OR 'venezuela' OR 'equatorial guinea':ab,ti,kw(7795960)<br>51. (#49 OR #50(7848037)<br>52. (#17 AND #27 AND #46 AND #51(1045)<br>53. (#52 AND [embase]/lim NOT ([embase]/lim AND [medline]/lim)(386)

	54. (#52 AND [embase]/lim NOT ([embase]/lim AND [medline]/lim) AND (2015:py OR 2016:py OR 2017:py OR 2018:py OR 2019:py)(157)
**Records retrieved**	157

**Search strategy**	
**Database**	JSTOR
**Date**	04-11-2019
**Publication date restrictions**	Last 5 years
**Language restrictions**	none
**Other limits**	none
**Search strategy**	((ab:((maternal or perinatal or postnatal or postpartum or prenatal) AND (service$ OR care)) AND (integrated care or coordinated care)) AND (middle income countries OR low income countries))
**Records retrieved**	2

Appendix 2. List of excluded studies and reasons for exclusion

Author	Year	Title	Reason for exclusion
Abejirinde et al.	2018	Mobile health and the performance of maternal health care workers in low- and middle-income countries: A realist review	Does not include relevant outcomes
Abejirinde et al.	2018	Unveiling the black box of diagnostic and clinical decision support systems for antenatal care: Realist evaluation	Intervention is not a maternal care integration strategy.
Afrizal et al.	2020	Evaluation of integrated antenatal care implementation in primary health care: A study from an urban area in Indonesia	No evaluation of integrated care strategy results.
Agustina et al.	2019	Universal health coverage in Indonesia: concept, progress, and challenges	Intervention is not a maternal care integration strategy.
Ahmed et al.	2016	Analyse transnationale des stratégies pour accomplir des progrès en vue des objectifs mondiaux pour la santé de la femme et de l’enfant	Does not include relevant outcomes
Aiga et al.	2018	Cost savings through implementation of an integrated home-based record: a case study in Vietnam	Does not include relevant outcomes
Akrami et al.	2012	Mothers' knowledge, attitude, and practice: The performance of the nationwide integrated maternal health care project	Does not include relevant outcomes
Akter et al.	2016	Workforce Interventions to Deliver Postnatal Care to Improve Neonatal Outcomes in	Intervention is not a maternal care integration strategy.

Author	Year	Title	Reason for exclusion
		Low- and Lower-Middle-Income Countries: A Narrative Synthesis	
Alam et al.	2019	Patients' and doctors' perceptions of a mobile phone-based consultation service for maternal, neonatal, and infant health care in Bangladesh: A mixed-methods study	No evaluation of integrated care strategy results
Alves et al.	2020	Advances in obstetric telemonitoring: a systematic review	Not middle-income and low-income countries
Atnafu et al.	2016	Can a community-based maternal care package in rural Ethiopia increase the use of health facilities for childbirth and reduce the stillbirth rate?	Does not include relevant outcomes
Badr et al.	2013	Renforcer les ressources humaines pour la santé par la mise en place de dispositifs d'information, de coordination et de responsabilisation: Le cas du Soudan	Intervention is not a maternal care integration strategy.
Baqui et al.	2008	Effect of community-based newborn-care intervention package implemented through two service-delivery strategies in Sylhet district, Bangladesh: a cluster-randomised controlled trial	Does not include relevant outcomes
Belizán et al.	2011	Stages of change: A qualitative study on the implementation of a perinatal audit programme in	Intervention is not a maternal care integration strategy.

Author	Year	Title	Reason for exclusion
		South Africa	
Bennett et al.	2020	Modelling cost benefit of community-oriented primary care in rural South Africa	Does not include relevant outcomes
Bergh et al.	2014	Implementing facility-based kangaroo mother care services: Lessons from a multi-country study in Africa	Intervention is not a maternal care integration strategy.
Bhutta et al.	2008	Alma-Ata: Rebirth and Revision 6 Interventions to address maternal, newborn, and child survival: what difference can integrated primary health care strategies make?	Does not include relevant outcomes
Bitton et al.	2017	Primary Health Care as a Foundation for Strengthening Health Systems in Low- and Middle-Income Countries	Study type does not meet eligibility criteria.
Blank et al.	2013	quality of prenatal and maternal care: Bridging the know-do gap (QUALMAT study): An electronic clinical decision support system for rural Sub-Saharan Africa	No evaluation of integrated care strategy results
Bonfrer et al.	2014	The effects of performance incentives on the utilisation and quality of maternal and child care in Burundi	Intervention is not a maternal care integration strategy.
Boone et al.	2016	Effects of community health interventions on under-5 mortality in rural Guinea-Bissau (EPICS): A cluster-randomised controlled	Does not include relevant outcomes

Author	Year	Title	Reason for exclusion
		trial	
Brown et al.	2019	Improving maternal and newborn health in rural Malawi: impact of a 3-year integrated approach of demand and supply side interventions using a repeated cross-sectional study	Publication type (conference poster, abstract)
Byrne et al.	2011	How the integration of traditional birth attendants with formal health systems can increase skilled birth attendance	Systematic literature review. Included publications were assessed for inclusion.
Canavan et al.	2017	Services de santé maternelle et néonatale en Éthiopie: évaluation et amélioration de la qualité	Intervention is not a maternal care integration strategy.
Cancelado et al.	2016	The Zuellig Family Foundation: A Strategic Philanthropic Approach to Integrated Care	Publication type: conference abstract
Chakrabarti et al.	2019	India's integrated child development services programme; equity and extent of coverage in 2006 and 2016	Does not include relevant outcomes
Chandhiok et al.	2015	Task-shifting challenges for provision of skilled birth attendance: A qualitative exploration	No evaluation of integrated care strategy results
Chia et al.	2016	The Zuellig Family Foundation: A Strategic Philanthropic Approach to Integrated Care	Publication type: conference abstract
Ciapponi et al.	2017	Delivery arrangements for health systems in low-income countries: An overview of systematic	Systematic literature review. Included publications were assessed for inclusion.

Author	Year	Title	Reason for exclusion
		reviews	
Cooper et al.	2016	Coming of age? Women's sexual and reproductive health after twenty-one years of democracy in South Africa	Intervention is not a maternal care integration strategy.
Daviaud et al.	2017	Overview, methods and results of multi-country community-based maternal and newborn care economic analysis	Does not include relevant outcomes
De Jongh et al.	2016	Barriers and enablers to integrating maternal and child health services to antenatal care in low and middle income countries	Intervention is not a maternal care integration strategy.
de Jongh et al.	2016	Integration of antenatal care services with health programmes in low- and middle- income countries: Systematic review	Systematic literature review. Included publications were assessed for inclusion.
Deshmukh et al.	2020	Utilisation of Postnatal Healthcare Services Delivered through Home Visitation and Health Facilities for Mothers and Newborns: An Integrative Review from Developing Countries	Intervention is not a maternal care integration strategy.
Diadjeng et al.	2018	Organizational context and leadership in the integration role of health care provider of integrated antenatal care team in public health centre	Intervention is not a maternal care integration strategy.
Dias et al.	2019	PREPARE: Protocol for a stepped wedge trial to evaluate whether a risk stratification model	Protocol

Author	Year	Title	Reason for exclusion
		can reduce preterm deliveries among women with suspected or confirmed preterm pre-eclampsia	
Doan et al.	2018	Utilization of Services Provided by Village-Based Ethnic Minority Midwives in Vietnam: Lessons From Implementation Research	Intervention is not a maternal care integration strategy.
Dolo et al.	2016	Libéria: Formation de sages-femmes aux soins obstétricaux avancés	Intervention is not a maternal care integration strategy.
Dudley et al.	2009	Strategies for integrating primary health services in middle and low-income countries at the point of delivery	Intervention is not a maternal care integration strategy.
Dynes et al.	2011	Home-based life saving skills in Matlab, Bangladesh: A process evaluation of a community-based maternal child health programme	Intervention is not a maternal care integration strategy.
Ekirapa-Kiracho et al.	2017	Uganda Newborn Study (UNEST) trial: Community-based maternal and newborn care economic analysis	Does not include relevant outcomes
El Arifeen et al.	2013	Community-based approaches and partnerships: Innovations in health-service delivery in Bangladesh	Intervention is not a maternal care integration strategy.
Franco et al.	2011	Effectiveness of collaborative improvement: Evidence from 27 applications in 12 less-developed and middle-income countries	Does not include relevant outcomes
Freeman et	2017	Comprehensive review of the	Intervention is not a maternal

Author	Year	Title	Reason for exclusion
al.		evidence regarding the effectiveness of community-based primary health care in improving maternal, neonatal and child health: 4. child health findings	care integration strategy.
Frøen et al.	2016	eRegistries: Electronic registries for maternal and child health	Intervention is not a maternal care integration strategy.
Gera et al.	2016	Integrated management of childhood illness (IMCI) strategy for children under five	Does not include relevant outcomes
Glenton et al.	2013	Barriers and facilitators to the implementation of lay health worker programmes to improve access to maternal and child health: Qualitative evidence synthesis	No evaluation of integrated care strategy results
Gopalan et al.	2012	Addressing maternal healthcare through demand side financial incentives: Experience of Janani Suraksha Yojana program in India	Intervention is not a maternal care integration strategy.
Graven et al.	2012	Achievement of the 2015 Millennium Development Goal 5A (Maternal Mortality) by Belize in 2011	Does not include relevant outcomes
Gyaltsen et al.	2014	Reducing high maternal mortality rates in western China: A novel approach	Intervention is not a maternal care integration strategy.
Hackett et al.	2018	Impact of smartphone-assisted prenatal home visits on women's use of facility delivery: Results	Does not include relevant outcomes

Author	Year	Title	Reason for exclusion
		from a cluster-randomized trial in rural Tanzania	
Hendricks et al.	2019	Tracking progress on the health status and service delivery outcomes for neonates and children in the metro west geographic service area of the cape metropole, 2010 - 2015	Intervention is not a maternal care integration strategy.
Huq et al.	2015	Effect of an integrated maternal health intervention on skilled provider's care for maternal health in remote rural areas of Bangladesh: A pre and post study	Does not include relevant outcomes
Hussein et al.	2012	The effectiveness of emergency obstetric referral interventions in developing country settings: A systematic review	Systematic literature review. Included publications were assessed for inclusion.
Hynes et al.	2017	Using a quality improvement approach to improve maternal and neonatal care in North Kivu, Democratic Republic of Congo	No evaluation of integrated care strategy results
Ilozumba et al.	2018	The Effect of a Community Health Worker Utilized Mobile Health Application on Maternal Health Knowledge and Behavior: A Quasi-Experimental Study	Intervention is not a maternal care integration strategy.
Isrctn et al.	2018	Strengthening reproductive, maternal, newborn and child health services in Bangladesh with an electronic health registry	Protocol

Author	Year	Title	Reason for exclusion
Iyer et al.	2017	Adapting maternal health practice to co-morbidities and social inequality: A systematic approach	Study type does not meet eligibility criteria.
Jacobs et al.	2012	Building on community outreach for childhood vaccination to deliver maternal and child health services in Laos: A feasibility assessment	Intervention is not a maternal care integration strategy.
Jiang et al.	2015	Task shifting of traditional birth attendants in rural China: a qualitative study of the implementation of institution-based delivery policy	Publication type (conference poster, abstract)
Jokhio et al.	2005	An intervention involving traditional birth attendants and perinatal and maternal mortality in Pakistan	Date of publication does not meet eligibility criteria.
Kadetz et al.	2011	Assumptions of global beneficence: Health-care disparity, the WHO and the outcomes of integrative health-care policy at local levels in the Philippines	Intervention is not a maternal care integration strategy.
Kaewkungwal et al.	2010	Application of smart phone in "better Border Healthcare Program": A module for mother and child care	Does not include relevant outcomes
Kara et al.	2017	The better birth program: Pursuing effective adoption and sustained use of the WHO safe childbirth checklist through coaching-based implementation	Intervention is not a maternal care integration strategy.

Author	Year	Title	Reason for exclusion
		in Uttar Pradesh, India	
Karanja et al.	2018	Factors influencing deliveries at health facilities in a rural Maasai Community in Magadi sub-County, Kenya	Intervention is not a maternal care integration strategy.
Kc et al.	2011	Developing community-based intervention strategies and package to save newborns in Nepal	Intervention is not a maternal care integration strategy.
Kebede et al.	2019	Effect of enhanced reminders on postnatal clinic attendance in Addis Ababa, Ethiopia: a cluster randomized controlled trial	Does not include relevant outcomes
Kelly et al.	2010	The role of a maternity waiting area (MWA) in reducing maternal mortality and stillbirths in high-risk women in rural Ethiopia	Does not include relevant outcomes
Kikuchi et al.	2018	Interventions integrating non-communicable disease prevention and reproductive, maternal, newborn, and child health: A systematic review	Does not include relevant outcomes
Kim et al.	2017	Understanding the role of intersectoral convergence in the delivery of essential maternal and child nutrition interventions in Odisha, India: A qualitative study	Intervention is not a maternal care integration strategy.
Kruk et al.	2014	Big push' to reduce maternal mortality in Uganda and Zambia enhanced health systems but lacked a sustainability plan	Intervention is not a maternal care integration strategy.

Author	Year	Title	Reason for exclusion
Kung'u et al.	2018	Design and implementation of a health systems strengthening approach to improve health and nutrition of pregnant women and newborns in Ethiopia, Kenya, Niger, and Senegal	No evaluation of integrated care strategy results
La Vincente et al.	2013	Supporting local planning and budgeting for maternal, neonatal and child health in the Philippines	Intervention is not a maternal care integration strategy.
Labrique et al.	2012	Pregnancy registration systems can enhance health systems, increase accountability and reduce mortality	Study type does not meet eligibility criteria.
Lassi et al.	2015	Community-based intervention packages for reducing maternal and neonatal morbidity and mortality and improving neonatal outcomes	Intervention is not a maternal care integration strategy.
Lassi et al.	2019	Community-based maternal and newborn educational care packages for improving neonatal health and survival in low- and middle-income countries	Intervention is not a maternal care integration strategy.
Leader et al.	2017	Collaborative implementation strategy for newborn resuscitation and essential care training in the Dominican Republic	Intervention is not a maternal care integration strategy.
Limato et al.	2019	What factors do make quality improvement work in primary health care? Experiences of maternal health quality	Intervention is not a maternal care integration strategy.

Author	Year	Title	Reason for exclusion
		improvement teams in three Puskesmas in Indonesia	
Lindgren et al.	2011	Using mobile clinics to deliver HIV testing and other basic health services in rural Malawi	Does not include relevant outcomes
Lund et al.	2012	Mobile phones as a health communication tool to improve skilled attendance at delivery in Zanzibar: A cluster-randomised controlled trial	Intervention is not a maternal care integration strategy
Mansour et al.	2010	Scaling up proven public health interventions through a locally owned and sustained leadership development programme in rural Upper Egypt	Does not include relevant outcomes
Martins et al.	2014	Implementing what works: A case study of integrated primary health care revitalisation in Timor-Leste	No evaluation of integrated care strategy results
Mbuagbaw et al.	2015	Health system and community level interventions for improving antenatal care coverage and health outcomes	Does not include relevant outcomes
McConnell et al.	2016	Can a community health worker administered postnatal checklist increase health-seeking behaviors and knowledge?: Evidence from a randomized trial with a private maternity facility in Kiambu County, Kenya	Intervention is not a maternal care integration strategy.
McDougal et al.	2016	Linkages Within the Reproductive and Maternal Health Continuum	Intervention is not a maternal care integration strategy.

Author	Year	Title	Reason for exclusion
		of Care in Bangladesh	
McNabb et al.	2015	Assessment of the quality of antenatal care services provided by health workers using a mobile phone decision support application in northern Nigeria: A pre/post-intervention study	Does not include relevant outcomes
Mensah et al.	2015	Impact of an electronic clinical decision support system on workflow in antenatal care: The QUALMAT eCDSS in rural health care facilities in Ghana and Tanzania	Does not include relevant outcomes
More et al.	2017	Community resource centres to improve the health of women and children in informal settlements in Mumbai: a cluster-randomised, controlled trial	Intervention is not a maternal care integration strategy.
Mori et al.	2015	The Maternal and Child Health (MCH) handbook in Mongolia: A cluster-randomized, controlled trial	Does not include relevant outcomes
Mothupi et al.	2020	Improving the validity, relevance and feasibility of the continuum of care framework for maternal health in South Africa: A thematic analysis of experts' perspectives	Intervention is not a maternal care integration strategy.
Mullany et al.	2010	Impact of community-based maternal health workers on coverage of essential maternal health interventions among internally displaced communities	Does not include relevant outcomes

Author	Year	Title	Reason for exclusion
		in eastern Burma: The MOM project	
Mushi et al.	2010	Effectiveness of community based safe motherhood promoters in improving the utilization of obstetric care. The case of Mtwara Rural District in Tanzania	Intervention is not a maternal care integration strategy.
Naimoli et al.	2015	Strategic partnering to improve community health worker programming and performance: Features of a community-health system integrated approach	Intervention is not a maternal care integration strategy.
Najafizada et al.	2014	Community health workers of Afghanistan: A qualitative study of a national program	Intervention is not a maternal care integration strategy.
Nct et al.	2017	Afya Credit Incentives for Improved Maternal and Child Health Outcomes in Kenya	Protocol
Nct et al.	2020	Patient-centered Mobile Technology Interventions to Improve Maternal Health in Uganda	Protocol
Neggers et al.	2013	Low birth weight outcomes: Why better in Cuba than Alabama?	Does not include relevant outcomes
Nkonki et al.	2017	A systematic review of economic evaluations of CHW interventions aimed at improving child health outcomes	Does not include relevant outcomes
Nyamtema et al.	2011	Maternal health interventions in resource limited countries: A	Intervention is not a maternal care integration strategy.

Author	Year	Title	Reason for exclusion
		systematic review of packages, impacts and factors for change	
Nyangara et al.	2018	Assessment of data quality and reporting systems for underserved populations: The case of integrated community case management programs in Nigeria	Does not include relevant outcomes
Osaki et al.	2019	Maternal and Child Health Handbook use for maternal and child care: A cluster randomized controlled study in rural Java, Indonesia	Intervention is not a maternal care integration strategy.
Osaki et al.	2019	Adapting home-based records for maternal and child health to users' capacities	Study type does not meet eligibility criteria.
Osrin et al.	2010	Perinatal interventions and survival in resource-poor settings: Which work, which don't, which have the jury out?	Does not include relevant outcomes
Oyeyemi et al.	2014	Giving cell phones to pregnant women and improving services may increase primary health facility utilization: A case-control study of a Nigerian project	Intervention is not a maternal care integration strategy.
Perry et al.	2017	Comprehensive review of the evidence regarding the effectiveness of community-based primary health care in improving maternal, neonatal and child health: 1. rationale, methods and database description	Does not include relevant outcomes

Author	Year	Title	Reason for exclusion
Pesec et al.	2017	Primary health care that works: The Costa Rican experience	Intervention is not a maternal care integration strategy.
Pfeiffer et al.	2019	Building health system capacity to improve maternal and newborn care: A pilot leadership program for frontline staff at a tertiary hospital in Ghana	Intervention is not a maternal care integration strategy.
Pradhan et al.	2011	Fitting Community Based Newborn Care Package into the health systems of Nepal	No evaluation of integrated care strategy results
Prinja et al.	2017	Impact of m-health application used by community health volunteers on improving utilisation of maternal, new-born and child health care services in a rural area of Uttar Pradesh, India	Intervention is not a maternal care integration strategy.
Pyone et al.	2014	Changing the role of the traditional birth attendant in Somaliland	No evaluation of integrated care strategy results
Rahimzai et al.	2014	Engaging frontline health providers in improving the quality of health care using facility-based improvement collaboratives in Afghanistan: Case study	Intervention is not a maternal care integration strategy.
Rahman et al.	2011	Effectiveness of an integrated approach to reduce perinatal mortality: Recent experiences from Matlab, Bangladesh	Does not include relevant outcomes
Ramaswamy et al.	2015	Transforming Maternal and Neonatal Outcomes in Tertiary Hospitals in Ghana: An	Intervention is not a maternal care integration strategy.

Author	Year	Title	Reason for exclusion
		Integrated Approach for Systems Change	
Ramsey et al.	2013	The Tanzania Connect Project: A cluster-randomized trial of the child survival impact of adding paid community health workers to an existing facility-focused health system	Protocol
Rao et al.	2018	India's integrated child development services scheme: challenges for scaling up	Intervention is not a maternal care integration strategy.
Ritika et al.	2019	Wearable sensors: A step towards smart monitoring of high risk pregnancies	Intervention is not a maternal care integration strategy.
Rivera-Romero et al.	2018	Mobile health solutions for hypertensive disorders in pregnancy: Scoping literature review	No evaluation of integrated care strategy results
Roman et al.	2014	Moving malaria in pregnancy programs from neglect to priority: Experience from Malawi, Senegal, and Zambia	No evaluation of integrated care strategy results
Rudrum et al.	2016	Understanding the meaning and role of gifts given to Ugandan mothers in maternity care settings: 'The help they give when they've seen how different you are'	Intervention is not a maternal care integration strategy.
Sacks et al.	2017	Comprehensive review of the evidence regarding the effectiveness of community-based primary health care in	Intervention is not a maternal care integration strategy.

Author	Year	Title	Reason for exclusion
		improving maternal, neonatal and child health: 3. neonatal health findings	
Sakeah et al.	2014	Can community health officer-midwives effectively integrate skilled birth attendance in the community-based health planning and services program in rural Ghana?	Intervention is not a maternal care integration strategy.
Sami et al.	2018	Understanding health systems to improve community and facility level newborn care among displaced populations in South Sudan: A mixed methods case study	No evaluation of integrated care strategy results
Sarfraz et al.	2014	Challenges in delivery of skilled maternal care - experiences of community midwives in Pakistan	Intervention is not a maternal care integration strategy.
Schaeffer et al.	2019	Development and evaluation of a mobile application for case management of small and sick newborns in Bangladesh	Intervention is not a maternal care integration strategy.
Schiffman et al.	2010	Community-Based Intervention Packages for Improving Perinatal Health in Developing Countries: A Review of the Evidence	Does not include relevant outcomes
Scott et al.	2018	What do we know about community-based health worker programs? A systematic review of existing reviews on community health workers	Intervention is not a maternal care integration strategy.

Author	Year	Title	Reason for exclusion
Sensalire et al.	2019	Saving mothers, giving life approach for strengthening health systems to reduce maternal and newborn deaths in 7 scale-up districts in northern Uganda	Does not include relevant outcomes
Shaikh et al.	2014	Emerging role of traditional birth attendants in mountainous terrain: A qualitative exploratory study from Chitral district, Pakistan	Intervention is not a maternal care integration strategy.
Shija et al.	2011	Maternal health in fifty years of Tanzania independence: Challenges and opportunities of reducing maternal mortality	Intervention is not a maternal care integration strategy.
Singh et al.	2016	Countdown to 2015 country case studies: Systematic tools to address the "black box" of health systems and policy assessment	Does not include relevant outcomes
Singh et al.	2017	iNICU – Integrated Neonatal Care Unit: Capturing Neonatal Journey in an Intelligent Data Way	Intervention is not a maternal care integration strategy.
Sondaal et al.	2016	Assessing the effect of mHealth interventions in improving maternal and neonatal care in low- And middle-income countries: A systematic review	Systematic literature review. Included publications were assessed for inclusion.
Srofenyoh et al.	2016	Measuring the impact of a quality improvement collaboration to decrease maternal mortality in a Ghanaian regional hospital	Does not include relevant outcomes

Author	Year	Title	Reason for exclusion
Storeng et al.	2016	lives in the balance: The politics of integration in the Partnership for Maternal, Newborn and Child Health	Does not include relevant outcomes
Sundari Ravindran et al.	2011	Are social franchises contributing to universal access to reproductive health services in low-income countries?	Study type does not meet eligibility criteria.
Thapa et al.	2019	The power of peers: An effectiveness evaluation of a cluster-controlled trial of group antenatal care in rural Nepal	Intervention is not a maternal care integration strategy.
Tomlinson et al.	2014	Goodstart: A cluster randomised effectiveness trial of an integrated, community-based package for maternal and newborn care, with prevention of mother-to-child transmission of HIV in a South African township	Does not include relevant outcomes
Tuominen et al.	2016	Exploratory analysis of the impact of quality management practices in a provider network services mother child health care in a middle-income economy	Publication type: conference abstract
Unger et al.	2018	Short message service communication improves exclusive breastfeeding and early postpartum contraception in a low- to middle-income country setting: a randomised trial	Does not include relevant outcomes
Var et al.	2015	Newborn Infection Control and Care Initiative for health facilities	Protocol

Author	Year	Title	Reason for exclusion
		to accelerate reduction of newborn mortality (NICCI): Study protocol for a randomized controlled trial	
Waiswa et al.	2015	Designing for action: Adapting and implementing a community-based newborn care package to affect national change in Uganda	Intervention is not a maternal care integration strategy.
Waiswa et al.	2012	The Uganda Newborn Study (UNEST): An effectiveness study on improving newborn health and survival in rural Uganda through a community-based intervention linked to health facilities - study protocol for a cluster randomized controlled trial	Protocol
Wallace et al.	2009	Integration of immunization services with other health interventions in the developing world: What works and why? Systematic literature review	Date of publication does not meet eligibility criteria.
Wallace et al.	2012	Experiences integrating delivery of maternal and child health services with childhood immunization programs: Systematic review update	Intervention is not a maternal care integration strategy.
Watkins et al.		Evaluation of the Information and Communications Technology for Maternal Newborn and Child Health Project (Chipatala cha pa Foni)	Intervention is not a maternal care integration strategy.
Werdenberg	2018	Successful implementation of a	Intervention is not a maternal

Author	Year	Title	Reason for exclusion
et al.		combined learning collaborative and mentoring intervention to improve neonatal quality of care in rural Rwanda 11 Medical and Health Sciences 1117 Public Health and Health Services	care integration strategy.
Willcox et al.	2019	Mobile technology for community health in Ghana: Is maternal messaging and provider use of technology cost-effective in improving maternal and child health outcomes at scale?	Does not include relevant outcomes
World Health et al.	2008	Country Case Study.Pakistan´s Lady Health Workers Programme. GHWA Task Force on Scaling Up Education and Training for Health Workers	Date of publication does not meet eligibility criteria.
Young et al.	2019	Integrating HIV, syphilis, malaria and anaemia point-of-care testing (POCT) for antenatal care at dispensaries in western Kenya: Discrete-event simulation modelling of operational impact	Does not include relevant outcomes
Zaman et al.	2011	Working toward decreasing infant mortality in developing countries through change in the medical curriculum	No evaluation of integrated care strategy results
Zimba et al.	2012	Newborn survival in Malawi: A decade of change and future implications	Does not include relevant outcomes

Appendix 3. Study characteristics

Authors	***Title***	***Year***	***Type of study***	***Country***	***LIC vs MIC***	***Aim***	***Conclusions***
Kearns et al.	Antenatal and postnatal care: A review of innovative models for improving availability, accessibility, acceptability and quality of services in low-resource settings	2016	Case studies	Ethiopia, Nepal,Pakistan, Tanzania, Bangladesh, Kenya, Malawi. (USA, Netherlands and Australia)	LIC	This study focused on identifying and analysing innovative approaches aimed at improving ANC and PNC and drawing potentially generalisable lessons from them.	Close monitoring of ANC and PNC quality and delivery models, health workforce support, appropriate use of electronic technologies, integrated care, a woman-friendly perspective, and adequate infrastructure are key elements of successful programmes that benefit the health and wellbeing of women, their newborns and families. However, a full evaluation of care delivery models is needed to establish their acceptability, accessibility, availability and quality.
Meyer et al.	Why high tech needs high touch: Supporting continuity of community primary health care	2018	Mixed-methods study	South Africa	MIC	This article seeks to understand how antenatal care users in a public facility in South Africa access care: the benefits and challenges of linking them to a CHW on household level through the use of AitaHealthTM. It also explores the obstacles and opportunities of using technology in a primary and community care setting and the learning experience in practice to ensure successful	Patient health data available to a health worker on a smartphone as part of COPC improve patient traceability and follow-up at home making timely referral possible. Health system developments that support patient care on community level could strengthen patient health access and overall health.

Authors	Title	Year	Type of study	Country	LIC vs MIC	Aim	Conclusions
						implementation.	
Dillip et al.	Can formalizing links among community health workers, accredited drug dispensing outlet dispensers, and health facility staff increase their collaboration to improve prompt access to maternal and child care? A qualitative study in Tanzania	2017	Qualitative	Tanzania	LIC	To explore barriers, successes, and promising approaches to increasing timely access to care by linking the three levels of health care provision.	The study highlights the benefits of approaches that link different levels of care providers to improve access to maternal and child health care. To strengthen this collaboration further, health campaign platforms should include retail drug dispensers as a type of community health care provider. To increase linkage sustainability, the council health management team needs to develop feasible supervision plans.
Kalita et al.	Role of innovative institutional structures in integrated governance: A case study of integrating health and nutrition programs in Chhattisgarh, India	2012	Mixed-methods study	India	MIC	The aim of this paper is to highlight the significance of integrated governance in bringing about community participation, improved service delivery, accountability of public systems and human resource rationalisation. It discusses the strategies of innovative institutional structures in translating such integration in the	The data indicate that integrated governance initiatives improved convergence between health and nutrition departments of the state at all levels. Also, innovative structures are important to implement the idea of integration, especially in contexts that do not have historical experience of such partnerships. Integration also contributed towards improved participation of communities in self-governance, community monitoring of government programs, and therefore, better services.

Authors	Title	Year	Type of study	Country	LIC vs MIC	Aim	Conclusions
						areas of public health and nutrition for poor communities.	
Balakrishnan et al.	Continuum of Care Services for Maternal and Child Health using mobile technology - a health system strengthening strategy in low and middle income countries	2016	Case study	India	MIC	To assess the effectiveness of the Continuum of Care Services (CCS) mHealth platform in terms of strengthening the delivery of maternal and child health (MCH) services in a district in Bihar, a resource-poor state in India	By virtue of its impact on quality, efficiency and equity of service delivery, health care manpower efficiency and governance, the mHealth inclusion at service provision level can be one of the potential strategies to strengthen the health system.
Borkum et al.	Evaluation of the Information and Communication Technology (ICT) Continuum of Care Services (CCS) Intervention in Bihar	2015	Mixed-methods study	India	MIC	To conduct a rigorous evaluation of the impacts of the Information and Communication Technology (ICT) Continuum of Care Services (CCS) intervention	The ICT intervention facilitated FLWs' interactions with households by helping them plan and coordinate their home visits, it led to substantial increases in the number of FLW-beneficiary interactions relative to the core Ananya package of interventions alone. Impacts on measures of visit quality—which the intervention could improve through features such as guided checklists for home visits and animated videos for changing behavior—were more mixed. We found impacts on beneficiary receipt of advice from FLWs on some specific topics (particularly related to infant feeding) but not on other topics, strong impacts on use of other Ananya job aid tools by FLWs, and no impacts on FLW visit duration. These improvements in the frequency and quality of visits were intended to translate into impacts on health behaviors, and we found substantive and significant effects on health behaviors in many—but not all—targeted domains. The findings of the study suggest that the use of this type of technology is a promising avenue to improve health outcomes in Bihar, and possibly more broadly. They

Authors	*Title*	*Year*	*Type of study*	*Country*	*LIC vs MIC*	*Aim*	*Conclusions*
							also suggest that the development of comprehensive mHealth tools like the ICT-CCS intervention, which integrates multiple features and addresses multiple health domains into a single tool (unlike many existing tools, which have a more narrow focus), may be a promising approach.
Jalloh-Vos Hermen Ormel et al.	Mobile health: Connecting managers, service providers and clients in Bombali district, Sierra Leone Mobile health: Connecting managers, service providers and clients in Bombali district, Sierra Leone Intervention study on mHealth for maternal and newborn	2014	Mixed-methods study	Sierra Leone	LIC	To assess the effect of integrating mobile communication strategies, as part of existing health service packages, on maternal and newborn health (MNH) service utilization in one health district, Bombali, in Sierra Leone.	The preliminary quantitative analysis and qualitative analysis suggest that the mHealth intervention relating to communication between health workers and clients may result in an increase in service utilization and better relationships between health workers and clients. This finding must be confirmed during the full analysis (when the data are made available).<br>The health information component of the mHealth intervention was appreciated by health workers and clients. However, the study could not demonstrate whether the health information needs of clients were sufficiently addressed. This issue needs to be further studied in order to adjust the service provided and meet the needs of clients.<br>The involvement of TBAs in health worker to client communication is a promising practice that is generally appreciated by all involved. TBAs can possibly play an important role as lynchpin in communication between clients and health workers.<br>Although no cost–benefit analysis was undertaken, a VPN is probably a very cost-efficient way to organize communication at health district level.<br>The inclusion of ambulance drivers in the VPN network appears to have improved the time needed for referral pick-up.<br>Maternal death notification improved considerably with the intervention. Data from this study show limited knowledge and relatively little use of the national phone line. This is not surprising due to the short period in which it has been accessible to the general public. Continued
Graven et al.	Decline in mortality with the	2013	Time series	Belize	MIC	To evaluate Belize health information system (BHIS)	

Authors	*Title*	*Year*	*Type of study*	*Country*	*LIC vs MIC*	*Aim*	*Conclusions*
	Belize Integrated Patient-Centred Country Wide Health Information System (BHIS) with Embedded Program Management					uptake by health care workers, and pre and post BHIS deployment mortality in selected areas and public health care expenditures	
Mwaniki et al.	Improving service uptake and quality of care of integrated maternal health services: The Kenya kwale district improvement collaborative	2014	Time series	Kenya	MIC	To explore whether improvement approaches can be applied to increase utilization of antenatal care (ANC), health facility deliveries, prevention of mother-to-child transmission services and adherence to ANC standards of care in a rural district in Kenya	Improvement approaches can be applied in rural health care facilities in low-income settings to increase utilization of services and adherence to standards of care. Using the quality improvement methodology to target integrated health services is feasible. Longer follow-up periods are needed to gather more evidence on the sustainability of quality improvement initiatives in low-income countries.
Osaki et al.	The role of home-based records in the establishment of a continuum of care for mothers, newborns, and children in Indonesia	2013	Cross-sectional study	Indonesia	MIC	This study aimed to identify the roles of home-based records both before and after childbirth, especially in provinces where the MCH handbook (MCHHB) was extensively promoted, by examining their association with MNCH service uptake	Our results suggest that pre- and post-natal home-based record use may be effective for ensuring service utilisation. In addition, since the handbook is an efficient home-based record for use throughout children's life courses, it could be an effective tool for promoting the continuum of MNCH care in Indonesia.
Orya et	Strengthening	2017	Qualitative	Sierra	LIC	To understand the	Our findings highlight the possible gains of the new roles and

Authors	***Title***	***Year***	***Type of study***	***Country***	***LIC vs MIC***	***Aim***	***Conclusions***
al.	close to community provision of maternal health services in fragile settings: An exploration of the changing roles of TBAs in Sierra Leone and Somaliland			Leone and Somaliland		perceptions of communities, stakeholder and TBAs themselves who have been trained in new roles to generate insights on strategies to engage with TBAs and to promote skilled birth attendance in fragile affected settings.	approaches for trained TBAs through further integrating them into the formal health system. Their potential is arguably critically important in promoting universal health coverage in fragile and conflict affected states (FCAS) where human resources are additionally constrained and maternal and newborn health care needs particularly acute.
Lhamsuren et al.	Taking action on the social determinants of health: Improving health access for the urban poor in Mongolia	2012	Qualitative	Mongolia	MIC	To present findings of an assessment of the implementation of the RED strategy, and, on the basis of this assessment, articulate lessons learned for equitable urban health planning.	Lessons from Mongolia mirror other international studies which point to the need to measure and take action on the social determinants of health at the local area level in order to adequately reduce persistent inequities in health care access for the urban poor.
Jiang et al.	Towards universal access to skilled birth attendance: The process of transforming the role of traditional birth attendants in Rural China	2016	Qualitative	China	MIC	This paper aims to demonstrate how TBAs in rural regions of China have been integrated into the health system under a policy of institutional delivery.	The China experience of transforming the role of TBAs in Guangxi rural area is an example of successfully engaging TBAs in promoting institution-based childbirth.
Carmichael et al.	Use of mobile technology by frontline health	2019	cluster-randomized controlled trial	India	MIC	To evaluate the impact of a novel mHealth tool that was implemented in Bihar,	Important improvements in FLW home visits and RMNCHN behaviors were achieved. The ICT-CCS tool shows promise for facilitating FLW effectiveness in improving RMNCHN behaviors

Authors	*Title*	*Year*	*Type of study*	*Country*	*LIC vs MIC*	*Aim*	*Conclusions*
	workers to promote reproductive, maternal, newborn and child health and nutrition: A cluster randomized controlled trial in Bihar, India					one of India's poorest and most populous states (104.1 million, 88.7% rural) which relies heavily on FLWs to provide community-level reproductive, maternal, newborn and child health and nutrition (RMNCHN)-related services	

Appendix 4 Quality appraisal

Case studies

Study		Kearns et al.	Balakrishnan et al.	Kalita et al.
Did the study address a clearly focused question / issue?	****Yes/ no/can´t tell***	Yes	Yes	Yes
Is the research method (study design) appropriate for answering the research question?	****Yes/no/can´t tell***	Yes	Yes	Yes
Are both the setting and the subjects representative with regard to the population to which the findings will be referred?	****Yes/no/can´t tell***	Yes	Yes	Can´t tell
Is the researcher's perspective clearly described and taken into account?	****Yes/no/can´t tell***	Can´t tell	Can´t tell	Can´t tell
Are the methods for collecting data clearly described?	****Yes/no/can´t tell***	Yes	Yes	No
Are the methods for analyzing the data likely to be valid and reliable? Are quality control measures used?	****Yes/no/can´t tell***	Yes	Yes	Can´t tell

Was the analysis repeated by more than one researcher to ensure reliability?	****Yes/no/can´t tell***	Can't tell	Can't tell	Can't tell
Are the results credible, and if so, are they relevant for practice?	****Yes/no/can´t tell***	Yes	Yes	Yes
Are the conclusions drawn justified by the results?	****Yes/no/can´t tell***	Yes	Yes	can´t tell
Are the findings of the study transferable to other settings?	****Yes/no/can´t tell***	Yes	Yes	Can´t tell
Overall score	***High, good, moderate, low, poor***	Good	Good	Moderate

Quantitative studies

Study		**Graven et al.**	**Mwaniki et al.**	**Osaki et al.**	**Carmichael et al.**
Does the study provide all the relevant background needed for an informed assessment?	****Yes/No/CD, cannot determine/ NA:not applicable/NR:not reported***	Yes	Yes	Yes	Yes
Was the research question or objective in this paper clearly stated?	****Yes/No/CD, cannot determine/ NA:not applicable/NR:not reported***	Yes	Yes	Yes	Yes

Study		**Graven et al.**	**Mwaniki et al.**	**Osaki et al.**	**Carmichael et al.**
Was the study population clearly specified and defined?	****Yes/No/CD, cannot determine/ NA:not applicable/NR:not reported***	Yes	Yes	Yes	Yes
Was a sample size justification, power description, or variance and effect estimates provided?	****Yes/No/CD, cannot determine/ NA:not applicable/NR:not reported***	NA	NA	NA	Yes
Are the individuals selected likely to be representative of the target population	****Very likely/ Somewhat likely/Not likey/Can't tell***	Yes	Yes	Yes	Yes
Data collection tools valid	****Yes/No/Can't tell***	Yes	Can't tell	Yes	Yes
Data collection tools reliable	****Yes/No/Can't tell***	Yes	Can't tell	No	Yes
Are the statistical methods appropriate for the study design	****Yes/No/Can't tell***	Yes	Yes	Yes	Yes
Overall score	***High, good, moderate, low, poor***	High	Moderate	Good	High

Qualittative studies

Reference		**Orya et al.**	**Lhamsuren et al.**	**Jiang et al.**	**Dillip, et al.**

Was there a clear statement of the aims of the research?	****Yes/No/Can't tell/NA***	Yes	Yes	Yes	Yes
Is a qualitative methodology appropriate?	****Yes/No/Can't tell/NA***	Yes	Yes	Yes	Yes
Was the research design appropriate to address the aims of the research?	****Yes/No/Can't tell/NA***	Yes	Yes	Yes	Yes
Was the recruitment strategy appropriate to the aims of the research?	****Yes/No/Can't tell/NA***	Yes	No	Yes	Yes
Was the data collected in a way that addressed the research issue?	****Yes/No/Can't tell/NA***	Yes	Can´t tell	Yes	Yes

Has the relationship between researcher and participants been adequately considered?	****Yes/No/Can't tell/NA***	Can´t tell	Can´t tell	Can´t tell	Can´t tell
Was the data analysis sufficiently rigorous?	****Yes/No/Can't tell/NA***	Yes	Can't tell	Yes	Yes
Is there a clear statement of findings?	****Yes/No/Can't tell/NA***	Yes	Yes	Yes	Yes
How valuable is the research?	***2: very valuable, 1: valuable 0: not very valuable***	2	1	2	2
Overall score	***High, good, moderate, low, poor***	High	Moderate	High	High

Mixed methods studies

Reference		**Meyer et al,**	**Borkum et al.**	**Jalloh-Vos et al.**

Does the study provide all the relevant background needed for an informed assessment?	****Yes/No/CD, cannot determine/ NA:not applicable/NR:not reported***	No	Yes	Yes
Was the research question or objective in this paper clearly stated?	****Yes/No/CD, cannot determine/ NA:not applicable/NR:not reported***	No	Yes	Yes
Was the study population clearly specified and defined?	****Yes/No/CD, cannot determine/ NA:not applicable/NR:not reported***	Yes	Yes	Yes
Was a sample size justification, power description, or variance and effect estimates provided?	****Yes/No/CD, cannot determine/ NA:not applicable/NR:not reported***	No	Yes	Yes
Are the individuals selected likely to be representative of the target population	****Very likely/ Somewhat likely/Not likey/Can't tell***	Somewhat likely	Yes	Yes
Data collection tools valid	****Yes/No/Can't tell***	Can´t tell	Yes	Yes

Data collection tools reliable	****Yes/No/Can't tell***	Can´t tell	Yes	Yes
Are the statistical methods appropriate for the study design	****Yes/No/Can't tell***	Yes	Yes	Yes
Was there a clear statement of the aims of the research?	****Yes/No/Can't tell/NA***	No	Yes	Yes
Is a qualitative methodology appropriate?	****Yes/No/Can't tell/NA***	Yes	Yes	Yes
Was the research design appropriate to address the aims of the research?	****Yes/No/Can't tell/NA***	Yes	Yes	Yes
Was the recruitment strategy appropriate to the aims of the research?	****Yes/No/Can't tell/NA***	Yes	Yes	Yes
Was the data collected in a way that addressed the research issue?	****Yes/No/Can't tell/NA***	Can´t tell	Yes	Yes
Has the relationship between researcher and participants been adequately considered?	****Yes/No/Can't tell/NA***	Can´t tell	Can´t tell	Can´t tell
Was the data analysis sufficiently rigorous?	****Yes/No/Can't tell/NA***	Yes	Yes	Yes
Is there a clear statement of findings?	****Yes/No/Can't tell/NA***	No	Yes	Yes
How valuable is the research?	***2: very valuable, 1: valuable 0: not very valuable***	1	2	2
Overall score	***High, good, moderate, low, poor***	Moderate	High	High
